# Lead-Binding Proteins: A Review

**DOI:** 10.1155/2011/686050

**Published:** 2011-09-19

**Authors:** Harvey C. Gonick

**Affiliations:** ^1^Division of Nephrology, David Geffen School of Medicine at UCLA, Los Angeles, CA, USA; ^2^Division of Nephrology, VA Medical Center West Los Angeles, Research Service 151, Building 114, Room 330, 11301 Wilshire Boulevards, Los Angeles, CA 90073, USA

## Abstract

Lead-binding proteins are a series of low molecular weight proteins, analogous to metallothionein, which segregate lead in a nontoxic form in several organs (kidney, brain, lung, liver, erythrocyte). Whether the lead-binding proteins in every organ are identical or different remains to be determined. In the erythrocyte, delta-aminolevulinic acid dehydratase (ALAD) isoforms have commanded the greatest attention as proteins and enzymes that are both inhibitable and inducible by lead. ALAD-2, although it binds lead to a greater degree than ALAD-1, appears to bind lead in a less toxic form. What may be of greater significance is that a low molecular weight lead-binding protein, approximately 10 kDa, appears in the erythrocyte once blood lead exceeds 39 **μ**g/dL and eventually surpasses the lead-binding capacity of ALAD. In brain and kidney of environmentally exposed humans and animals, a cytoplasmic lead-binding protein has been identified as thymosin **β**4, a 5 kDa protein. In kidney, but not brain, another lead-binding protein has been identified as acyl-CoA binding protein, a 9 kDa protein. Each of these proteins, when coincubated with liver ALAD and titrated with lead, diminishes the inhibition of ALAD by lead, verifying their ability to segregate lead in a nontoxic form.

## 1. Introduction

This paper focuses on lead-binding proteins, which, in contrast to the majority of articles in this issue, are not toxic compounds but rather low molecular weight proteins which have the capacity to bind lead and to sequester the lead in a nontoxic form. In the discussion which follows, careful note should be taken as to which lead-binding proteins are inducible, that is, increased in concentration after exposure to lead, and which are constitutive, that is, are present within the organism at all times and may have binding sites saturable by lead but no discernible increase in protein content. The second situation is perhaps most pertinent to enzymes inhibitable by lead, the first to proteins not normally present or present in low quantities in the organism but which increase after lead exposure and offer protection against lead toxicity. The history of lead-binding proteins can be dated back to 1936, when Blackman first described the presence of intranuclear inclusion bodies in the liver and kidney as manifestations of lead poisoning [1]. The next phase in this saga consisted of detailed studies of the composition of renal tubular intranuclear inclusion bodies and the consequent alterations in mitochondrial structure and function which followed, first by Goyer and colleagues, later by Shelton et al. [2–5], and Klann and Shelton [6, 7].

## 2. Lead-Binding Proteins within Intranuclear Inclusion Bodies in Kidney

Goyer [8] began his studies by examining the renal tubules of rats fed 1% lead acetate for up to 20 weeks, finding that dense, deeply staining intranuclear inclusions were located in the straight portion of the proximal tubules, accompanied by swollen, globular, or ovoid, closely packed mitochondria with many marginated, irregular, or vesicular cristae. Accompanying these mitochondrial changes was the presence of generalized aminoaciduria. In a subsequent paper in the same issue, Goyer and colleagues [9] isolated mitochondria from lead-exposed and control rats and demonstrated that mitochondria from the lead-exposed rats show reduced rates of respiration and oxidative phosphorylation.

 Lead within the kidneys in lead-poisoned rats was found to be concentrated in the nuclei and within nuclei in the nuclear inclusion body [10, 11]. Choie and Richter [12] showed further that rapid induction of inclusion bodies by injections of lead salts in the rat resulted in cytoplasmic inclusions, suggesting that they were precursors to the intranuclear inclusions. This was further confirmed by McLachlin et al. [13] who showed in tissue culture studies of rat kidney cells incubated with lead that the cytoplasmic inclusion bodies preceded and disappeared shortly after the appearance of nuclear inclusion bodies.

 Lead-containing nuclear inclusions were also found in organs other than the kidney, including liver and glial cells of the central nervous system [14]. Moore et al. [15] dissolved the rat renal intranuclear inclusions in strong denaturing agents and found that the protein in the inclusions is acidic, with high levels of aspartic acid, glutamic acid, glycine and cystine. Moore and Goyer [16] later characterized the protein as a 27.5 kDa protein, which migrates as a single band on acrylamide gel electrophoresis. Repeated intraperitoneal injections of calcium disodium ethylenediamine tetraacetic acid (CaNa_2_EDTA) resulted in disappearance of the inclusion bodies in lead-exposed rats, together with a marked decrease in kidney lead levels [17].

 Shelton and coworkers have also explored the composition of the lead-binding proteins in the nuclear inclusion proteins of lead-exposed rat kidneys. Shelton and Egle [2] first described a 32 kDa protein with an isoelectric point of 6.3, which was isolated from the kidneys of rats treated with 1% lead acetate in rat chow or 0.75% lead acetate in drinking water, given for 13–17 weeks. In contrast to the work of Goyer and coworkers, they employed two-dimensional gel electrophoresis to isolate the protein from the nuclear inclusion bodies and demonstrated that it was present in lead-exposed, but not control kidneys (hence inducible). This protein has been termed p32/6.3. Inhibitor studies with cycloheximide and actinomycin D [13, 18] had indicated earlier that protein synthesis was required for induction of the nuclear and cytoplasmic inclusion bodies.

 Egle and Shelton [3] then turned their attention to the brain, where they found unexpectedly that p32/6.3, now characterized by a monoclonal antibody, was constitutively present in cerebral cortex, both in neurons and astrocytes. The protein was concentrated in the insoluble nuclear protein, similar to the lead-exposed kidney. Brain p32/6.3 was detected in rat, mouse, dog, man and chicken. In rat brain, adult levels were achieved in 1-2 weeks after birth whereas only trace amounts were found at 3 days. Brain p32/6.3 increases between postnatal days 10–12 in the guinea pig and days 15–21 in the rat, suggesting that the increase may be related in part to exposure to the external environment [5]. When neuroblastoma cells were cultured after 1 and 3 days exposure to lead, the abundance of p32/6.3 increased. Simultaneous incubation with lead and cycloheximide or actinomycin D also showed an increase in p32/6.3, suggesting that lead selectively retards the degradation of the brain protein [6]. The amino acid composition of partially purified p32/6.3 revealed a high percentage of glycine, aspartic and glutamic acid [4]. Thus Shelton and coworkers have established that an inducible protein, p32/6.3, can be extracted from nuclear inclusion bodies from the lead-exposed rat kidney and a similar or identical protein from adult rat brain. Whether the brain protein is constitutive or inducible by exposure to environmental lead has yet to be determined.

 Selvin-Testa et al. [19] and Harry et al. [20] reported that developing rat brain astrocytes exposed to lead developed an elevation in glial fibrillary acidic protein (GFAP), a developmentally regulated protein. Harry et al. [20] consider that the elevated levels of GFAP mRNA during the second postnatal week after lead exposure may reflect the demand on astrocytes to sequester lead.

 Oskarsson and Fowler [21] examined the influence of pretreatment with lead by a single i.p. injection of lead acetate (50 mg lead per kg body wt) 1, 3 and 6 days before injecting ^203^Pb. Rats were sacrificed 24 hours later and the kidneys were examined both microscopically and for the distribution of ^203^Pb. At 3 days rat, kidneys displayed fibrillar cytoplasmic inclusions, but, at 6 days these inclusions were less prominent and intranuclear inclusions were observed. ^203^Pb uptake at 6 days was maximal in the purified nuclear fraction and in the nuclear inclusion bodies (7x and 20x control, resp.).

## 3. Cytoplasmic Lead-Binding Proteins in Kidney and Brain

The remaining studies of nonlead-stimulated cytoplasmic kidney and brain lead-binding proteins have been provided by Fowler and associates.

 The first study [22] reported on the lead-binding proteins in kidney postmitochondrial cytosolic fractions. Binding of ^203^Pb was found in two protein fractions of control kidneys with molecular weights of 11.5 and 63 kDa. Binding was markedly decreased after lead pretreatment. The use of cadmium to stimulate metallothionein synthesis did not increase ^203^Pb binding to the 11.5 kDa protein. The two binding proteins were also present in brain but not in liver or lung. Subsequently, Mistry et al. [23] demonstrated three lead-binding proteins (11.5 kDa, 63 kDa and >200 kDa) in rat kidney cytosol, which had binding characteristics of high-affinity low-capacity sites with respective *Kd* values of 13, 40, and 123 nM. The 11.5 kDa and possibly the 63 kDa protein were capable of translocating lead into the nucleus as shown by uptake of ^203^Pb into nuclei incubated with tagged cytosolic proteins. 

 Goering and Fowler [24] showed that the 11.5 kDa protein, but not the 63 kDa protein, was capable of reversing lead-induced ALAD inhibition in liver homogenates. This effect was mediated both by chelation of lead by the lead-binding protein and by donation of zinc to ALAD [25]. Various divalent metal ions influence the binding of lead to the rat kidney cytosolic binding proteins, with an order of displacement of Cd^++^  > Zn^++^  > Pb^++^. Ca^++^ had no effect, while Fe^++^ had a cooperative effect [26]. These observations may account for the previously demonstrated effect of concomitant lead and cadmium administration in reducing total kidney lead [27] and preventing the development of intranuclear inclusion bodies [28]. 

More recent studies by Fowler and DuVal [29] identified the rat renal lead-binding protein as a cleavage product of *α*2-microglobulin, with a *Kd* of 10^−8^ M lead. There are two forms of the protein in the kidney, differentiated by the cleavage of the first 9 N-terminal residues from the higher molecular weight form. Other studies by Smith and coworkers [30] have found two lead-binding proteins in environmentally exposed human kidneys, identified as acyl-CoA-binding protein (ACBP) or diazepan-binding inhibitor (molecular weight 9 kDa) and thymosin *β*4 (molecular weight 5 kDa). These polypeptides have a high-affinity for lead (*Kd*~14 nM).

 In rat brain, Goering et al. [31] and DuVal and Fowler [32] explored the effect of environmental lead on lead-binding proteins and the ability of rat brain lead-binding proteins to diminish the inhibition of hepatic ALAD by lead. In the first study, a brain protein of 12 kDa was described, in comparison to the kidney lead-binding protein of 9 kDa. Both competitions of lead binding between the brain lead-binding protein and ALAD and donation of zinc by the brain protein (shown by ^65^Zn uptake) were found to account for the decreased ALAD inhibition. In the second study the rat brain lead-binding protein was described as having a molecular weight of 23 kDa, with significant levels of glutamic acid, aspartic acid, and cysteine. Polyclonal antibody to rat renal lead-binding proteins showed a lack of reactivity with the brain protein, indicating that the proteins are immunologically distinct.

 Fowler et al. [33] examined monkey kidney and brain from nonlead treated animals and isolated lead-binding proteins that also had a relatively high content of aspartic and glutamic amino acid residues and were similar in size to the rat lead-binding proteins. Polyclonal antibodies to *α*2-microglobulin and metallothionein did not cross-react with either monkey kidney or brain proteins. In environmentally lead-exposed humans, Quintanilla-Vega et al. [34] subsequently isolated from brain a thymosin *β*4 and a second, as yet unidentified, protein with a molecular weight of 20 kDa and a pI of 5.9.

 As reported earlier, lead also binds to p32/6.3, a low-abundance, highly conserved nuclear matrix protein that becomes a prominent component of lead-induced intranuclear inclusion bodies [6]. Expression of this protein increases significantly during ontogeny, and was proposed as an indicator of neuronal maturation [7]. Expression also increases markedly in the presence of acute lead exposure *in vitro*, suggesting that lead either structurally alters the protein or inhibits a protease for which p32/6.3 is a substrate [5].

 Recently, an astroglial glucose-regulated protein (GRP78) has been identified that acts as a molecular chaperone in endoplasmic reticulum [35, 36]. Intracellular levels of this protein are increased in cultured astroglia during a 1-week exposure to lead. GRP78 depletion significantly increased the sensitivity of cultured glioma cells to lead, as indicated by the generation of reactive oxygen species. This suggests that GRP78 is a component of the intracellular tolerance mechanism that handles high intracellular lead accumulation. Thus it appears that lead directly targets the protein, enabling it to play a protective role in lead neurotoxicity. The generation of reactive oxygen species also has been reported to occur via lead binding to astroglial copper-transporting ATPase, resulting in disruption in copper homeostasis [37].

## 4. Lead-Binding Proteins in Erythrocytes

Intraerythrocytic lead binding was initially attributed primarily to hemoglobin, molecular weight 64 kDa [38–41], but more recent studies have ascribed the major lead binding to delta-aminolevulinic acid dehydratase (ALAD), molecular weight 240 kDa (vide infra), which is an important step in heme biosynthesis [42]. In contrast to this protein, several studies have focused on an inducible low molecular weight protein which appears in workers chronically exposed to lead and which seems to have a protective effect. The first recognition of this protein was by Raghavan and Gonick [41] who found an approximately 10 kDa protein in lead workers but not in controls following Sephadex G-75 fractionation. Upon subsequent SDS-polyacrylamide gel electrophoresis, the protein split into two bands, only the uppermost of which contained lead. Raghavan et al. [43] then went on to fractionate the erythrocyte lead into a “hemoglobin” fraction, 10 kDa fraction, free lead, and a “residual lead” fraction, thought to be composed of membrane lead and a high molecular weight fraction. Lead workers manifesting toxicity at both high blood lead and relatively low blood lead levels showed high levels of residual lead, attributed in the workers with toxicity at low blood lead levels to a very low quantity of the 10 kDa fraction. In a follow-up paper, Raghavan et al. [44] found elevated levels of lead in the high molecular weight fraction (prehemoglobin) and in the membrane fraction in workers with toxicity at both high and low blood leads. Again, those with toxicity at low blood lead had low levels of the lead bound to the 10 kDa protein. Membrane lead was found to correlate inversely with membrane Na-K-ATPase; no correlation was seen with total blood lead. Gonick et al. [45] partially purified the 10 kDa protein by HPLC using a protein I-125 column, followed by isoelectric focusing on a sucrose gradient column. Three protein peaks resulted, one of 30 kDa, and two of approximately10 kDa. Only one of the latter peaks contained lead. This peak had a pI of 5.3 and a molecular weight, determined by SDS-PAGE, of 12 kDa. The majority of lead was found in this peak, which also contained calcium, zinc, and cadmium. Amino acid analysis showed a very high percentage of glycine (44%), lower quantities of histidine, aspartate, and leucine. The presence of glycine and aspartate corresponds in part to the composition of amino acids in the intranuclear inclusion bodies, as described by Shelton et al. [4] and Moore et al. [15].

 Ong and Lee [40] studied the distribution of ^203^Pb in components of normal human blood. Ninety-four percent of ^203^Pb was incorporated into the erythrocyte, and 6% remained in the plasma. SDS-PAGE of plasma showed that 90% was present in the albumin fraction. Within the erythrocyte membrane the most important binding site was the high molecular weight fraction, about 130–230 kDa. Within the erythrocytic cytoplasm the protein band associated with ^203^Pb had a molecular weight of 67 kDa as shown by the elution characteristics on G-75 chromatography. This was thought to be hemoglobin. Lolin and O'Gorman [39] and Church et al. [46, 47], following the same procedure as Raghavan and Gonick [41], confirmed the findings of a low molecular weight protein in the erythrocytes of lead workers, not found in control patients. Lolin and O'Gorman [39] quantitated the protein, which ranged from 8.2 to 52.2 mg/L RBC in lead workers but none in controls, again implying an inducible protein. They found that the low molecular weight protein first appeared when the blood lead concentration exceeded 39 *μ*g/dL. A positive correlation was seen between the amount of the intraerythrocytic low molecular weight protein and dithiothreitol-activated activity (i.e., “restored” ALAD activity) but not the nonactivated activity. Church et al. [46, 47] also confirmed the findings of Raghavan and Gonick [41]. In the initial paper, they described two patients with high blood lead levels, an asymptomatic worker with a blood lead of 180 *μ*g/dL and a symptomatic worker with a blood lead of 161 *μ*g/dL. In the first patient, approximately 67% of the erythrocyte lead was bound to a low molecular weight protein of approximately 6-7 kDa. In the second patient, the protein only contained 22% of the total erythrocytic lead. In their remaining paper [47], Church et al found that a sample of the low molecular weight protein purified from lead workers, which they termed protein M, had characteristics of metallothionein, such as a molecular weight of 6.5 kDa, a pI between 4.7 and 4.9 and a greater UV absorbance at 254 nm than at 280 nm. Amino acid composition showed 33% cysteine but no aromatic amino acids. This composition differed from that of the low molecular weight protein described by Gonick et al. [45], which had a molecular weight of 12 kDa, a pI of 5.3, and amino acid analysis which showed no cysteine. This discrepancy might be explained by a combined lead and cadmium exposure in the Church et al. study, which may have produced a lead-thionein (vide infra).

 Xie et al. [48] used a Biogel A column instead of Sephadex G-75 to separate lead-binding proteins from erythrocyte hemolysates from a control patient and from lead-exposed workers. In this study they showed clearly that the major lead-binding occurred to a large molecular weight protein, consistent with ALAD, in both the controls and lead workers. When they added increasing amounts of lead to the blood of the control patient, a second, low molecular weight, lead-binding peak occurred which became larger than the lead binding to the ALAD peak when the lead concentration exceeded approximately 50 *μ*g/dL. This second peak was also seen in a chronically lead-exposed worker, and was estimated to be less than 30 k Da in molecular weight. Thus these results are consistent with the aforementioned studies.

## 5. Is ALAD an Inducible Enzyme and Is It the Principal Lead-Binding Protein in the Erythrocyte?

The enzyme ALAD has been found to be the most sensitive indicator of lead exposure and toxicity [49, 50]. In the 1980's, two articles were presented which appear to show that ALAD is inducible after lead exposure in humans. By comparing a nonexposed control population to lead workers, and assaying ALAD by means of immunoassay or as “restored” ALAD activity (i.e., incubation with heat, zinc and dithiothreitol), both articles indicated that the *amount* of ALAD, as contrasted to ALAD *activity*, was increased by lead exposure [51, 52]. Similar findings were reported for the rat [53]. Subsequent studies have focused on the effect of ALAD polymorphism on the susceptibility to lead intoxication. ALAD is a zinc-containing enzyme, which catalyzes the second step of heme synthesis, that is, catalyzes the condensation of two delta-aminolevulinic acid molecules into one molecule of porphobilinogen [51]. It is a polymorphic protein with three isoforms: ALAD-1, ALAD 1-2, and ALAD 2-2. Several studies have shown that, with the same exposure to lead, individuals with the ALAD-2 gene have higher blood lead levels [54–60]. Initially it was thought that these individuals might be more susceptible to lead poisoning [59], but it is now appreciated that the ALAD-2 gene offers protection against lead poisoning by binding lead more securely [61]. It has been shown that ALAD-2 is more electronegative than ALAD-1 and thus the ALAD-2 enzyme may have a higher affinity/stability for lead than ALAD-1 [62]. In support of this statement it can be cited that individuals with the ALAD 1-2/2-2 genotypes in comparison to those with ALAD 1-1 genotypes have not only higher blood lead but also decreased plasma aminolevulinic acid [63], lower zinc protoporphyrin [56], lower cortical bone lead [58], and lower amounts of dimercaptosuccinic-acid (DMSA-) chelatable lead [64]. 

 The significance of erythrocyte ALAD lead-binding was initially confirmed by a study by Bergdahl et al. [55], in which the authors used a FPLC Superdex 200 HR 10/30 chromatographic column coupled to ICP-MS (for determination of lead) to examine erythrocytes from lead workers and controls. They found the principal lead-binding protein peak to be of 240 kDa, rather than the presumed hemoglobin peak reported by Barltrop and Smith [38] and Raghavan and Gonick [41], using Sephadex G-75 chromatography. This was shown to be ALAD by binding to specific ALAD antibodies. Two additional smaller lead-binding peaks of 45 kDa and 10 kDa were also seen, but not identified. Bergdahl et al. [55] attributed the discrepancies in the studies to the fact that Sephadex G-75 separates proteins in the range of 3 to 80 kDa, making the separation of hemoglobin (molecular weight 64 kDa) from ALAD (molecular weight 240–280 kDa) very difficult. In addition, the earlier studies had utilized binding of ^203^Pb or ^210^Pb to identify the binding proteins, a technique which may have skewed the findings if ALAD were already saturated with lead. ALAD-binding capacity for lead has been measured at 85 *μ*g/dL in erythrocytes or 40 *μ*g/dl in whole blood [65], which would permit a greater degree of binding to the low molecular weight component when blood lead exceeded 40 *μ*g/dL. Bergdahl et al. [65] have speculated that the low molecular weight component might be acyl-CoA-binding protein, identical to the kidney lead-binding protein described by Smith et al. [30]. Goering and Fowler [66] had reported earlier that the presence of low molecular weight high-affinity (Kd 10^−8^ M) lead-binding proteins in kidney and brain served as protection against ALAD inhibition in those organs, whereas the absence of these low molecular weight proteins in liver contributed to the greater sensitivity to ALAD inhibition in that organ.

## 6. Lead-Binding Proteins in Rat Liver

Sabbioni and Marafante [67] explored the distribution of ^203^Pb in rat whole tissue as well as in subcellular fractions of liver. By far the largest quantity of lead recovered was in the kidney, with lesser amounts in liver, spleen, and blood. Upon subcellular fractionation of the liver, the majority of ^203^Pb was found in the nuclei, and most of the lead was detected in the nuclear membrane fraction bound exclusively to membrane proteins. The intranuclear lead was associated with histone fractions. As we have seen previously from Oskarsson et al. [22], lead-binding proteins were not found in the cytoplasm of the liver.

## 7. Lead-Binding Proteins in Intestine

 Fullmer et al. [68] have shown in the chick and cow that although lead does not stimulate lead-binding proteins in the intestine directly, lead can displace calcium from calcium-binding proteins, and thus calcium-binding proteins may play a role in intestinal lead transport. Purified calcium-binding protein from chick and cow, as well as calmodulin, troponin C, and oncomodulin, was dialyzed against added labelled and unlabelled lead or calcium. Results disclosed high-affinity binding sites, greater for lead than for calcium. Similar results were obtained with calmodulin, troponin C, and oncomodulin members of the troponin C super family of calcium-binding proteins.

## 8. Lead-Binding Proteins in Lung

Singh et al. [69] described intracellular lead-inclusion bodies in normal human lung small-airway epithelial cells cultured with either lead chromate particles or sodium chromate. Cells exposed to both forms of chromate underwent dose-dependent apoptosis. Lead-inclusion bodies were found in nucleus and cytoplasm of lead chromate, but not sodium chromate, treated cells. Lead, but not chromium, was detected in the inclusion bodies by energy dispersive X-ray analysis. The protein within the inclusion bodies has not been analyzed.

## 9. Relationship of Lead-Binding Protein to Metallothionein

Similarities of lead-binding protein to metallothionein have been discussed earlier. Maitani et al. [70] commented that hepatic zinc metallothionein could be induced by intravenous intraperitoneal injections of lead into mice, but not by subcutaneous injection. Ikebuchi et al. [71] found that a sublethal dose of lead acetate injected intraperitoneally into rats induced the synthesis of a lead metallothionein in addition to zinc metallothionein. The lead metallothionein contained 28% half cysteine and cross-reacted with an antibody against rat zinc-thionein II. 

 Goering and Fowler [66, 72] demonstrated that pretreatment of rats with zinc 48 and 24 hrs prior to injection of ^203^Pb resulted in both zinc and lead coeluting with a zinc-thionein fraction on Sephadex G-75 filtration. In addition, both purified zinc-thionein-I and II bound ^203^Pb *in vitro*. Gel filtration of incubates containing liver ALAD and ^203^Pb demonstrated that the presence of zinc-thionein alters the cytosolic-binding pattern of lead, with less bound to ALAD. Zinc-thionein also donates zinc to activate ALAD. In the second paper [66], Goering and Fowler found that pretreatment of rats with either cadmium or zinc affected liver ALAD activity when incubated with lead. Liver and kidney zinc-thioneins, and to a lesser extent, cadmium-zinc-thionein decreased the free pool of lead available to interact with ALAD, resulting in attenuated ALAD inhibition. Liu et al. [73] further showed that zinc-induced metallothionein in primary hepatocyte cultures protects against lead-induced cytotoxicity, as assessed by enzyme leakage and loss of intracellular potassium.

 Qu et al. [74] and Waalkes et al. [75] have shown that metallothionein-null phenotypic mice are more susceptible to lead injury over a 20-week period than wild-type mice. Unlike the wild-type mice, lead-treated metallothionein-null mice showed nephromegaly and significantly decreased renal function after exposure to lead. The metallothionein-null mice accumulated less renal lead than wild-type and formed no inclusion bodies. When the observations were extended to 104 weeks, renal proliferative lesions (adenoma and cystic tubular atypical hyperplasia) were more common and severe in metallothionein-null than in wild-type mice. A metastatic renal cell carcinoma occurred in a metallothionein-null mouse whereas none occurred in wild-type mice. Such studies lend credence to the view that metallothionein or a closely related gene is involved in the formation of lead-binding proteins in the kidney.

 A more recent study [76] further supports the involvement of metallothionein in lead-induced inclusion body formation. Immunochemical studies revealed that metallothionein could be detected on the outer surface of lead-induced renal inclusion bodies in wild-type mice. Cell lines from metallothionein-null and wild-type mice were exposed to lead, resulting in inclusion body formation in the wild-type, but not the metallothionein-null cells. When metallothionein was transfected into the metallothionein-null cells, inclusion bodies were seen. Further studies revealed that *α*-Synuclein, an aggresomal protein, showed poor basal expression in metallothionein-null cells and failed to increase after lead exposure, but increased rapidly in wild-type cells, then decreased after inclusion bodies were formed. An antibody pulldown assay confirmed a direct interaction between *α*-Synuclein and metallothionein as the proteins coprecipitated with an antibody to metallothionein.

## 10. Biochemical Studies of Metal Binding to Proteins

 Three important papers have appeared concerning the mechanism by which a metal ligand binds to proteins. Lead is known to displace physiologically relative metal ions, such as calcium and zinc, in proteins. Kirberger and Yang [77] reported that approximately 1/3 of the lead binding sites were identified as due to zinc or calcium ionic displacement, whereas two-thirds were opportunistic. Oxygen atoms from amino acids or water represent the major ligand for lead, followed by sulfur and nitrogen. Sulfur acts as the ligand in the case of displacement of zinc by lead at the zinc-binding sites in delta-aminolevulinic acid dehydratase (ALAD). Studies of calmodulin, a calcium binding protein with a propensity for displacement of calcium by lead, showed an initial activation followed by inhibition in response to increasing concentrations of lead. The latter was thought to result from more pronounced conformational changes resulting from additional opportunistic binding. Hanas et al. [78] explored whether the association of lead with chromatin might suggest that the deleterious effects may in part be mediated through alterations in gene function. They specifically examined whether lead altered DNA binding of cysteine-rich zinc finger proteins. It was found that inhibition of Cys_2_His_2_ zinc finger transcription factors by lead ions at concentrations near those known to have deleterious physiological effects was suggestive for a new molecular mechanism for lead toxicity. However, the changes were seen predominantly at lead concentrations varying from 100 to 400 ug/dL, above the industrial or pathophysiological range. Becker et al. [79] analyzed naturally occurring metal-binding proteins in rat liver and kidney, utilizing nondenaturing gel electrophoresis together with laser ablation inductively coupled plasma mass spectrometry (LA-ICP-MS). The mass range of proteins separated was from 45 to 120 kDa. Lead was found only in small quantities in the 65 kDa protein band, whereas zinc and copper were scattered throughout the protein bands. If this technique could be extended to the study of lead-treated as well as native rats and the protein mass separated down to 5 kDa, confirmation (or lack thereof) of the results reported earlier by Fowler and associates [22–24, 29, 33] might be anticipated.

## 11. Discussion

There appears to be a consensus that the enzyme, ALAD, a 240–280 kDa protein, is moderately inducible and is the major lead-binding protein within the erythrocyte. ALAD polymorphism influences the degree of lead binding as the ALAD-2 phenotype binds more lead in a nontoxic fashion than ALAD-1. What is more confusing is the nature and importance of the low molecular weight erythrocytic lead-binding protein. There is no doubt that it appears in lead-exposed workers once blood lead exceeds 40 *μ*g/dL but does not appear in controls. An intriguing observation was made by Xie et al. [48], who noted that the *in vitro* addition of lead to erythrocytes of controls resulted in the appearance of a low molecular weight lead-binding peak migrating in the same position as the low molecular weight peak from lead workers. This suggests that lead can effect a conformational change in a preexisting protein. The nature of the low molecular weight protein is also questionable as Gonick and colleagues [45] identified it as a 12 kDa protein with a high percentage of glycine, plus histidine, aspartic acid and leucine while Church and coworkers [46, 47] identified the protein as a 6.5 kDa molecule, with a large percentage of cysteine and a greater UV absorbance at 254 than 280 nm. These findings suggested to the latter authors that the protein might be a metallothionein.

 Metallothionein is a protein that is mildly inducible by lead, but to a much greater degree by zinc and cadmium. What is more significant is that lead binds to preformed metallothionein, stimulated by zinc or cadmium, so that under these conditions a lead-thionein is formed. If concomitant lead and cadmium exposure occurred in the lead workers described by Church et al. [46, 47], that could reasonably account for the finding of metallothionein in these workers. However the possible role of metallothionein as a component of the renal lead-binding protein seems more likely as metallothionein-null mice failed to respond to lead exposure by developing intranuclear lead inclusion bodies, whereas metallothionein-null cells transfected with metallothionein were capable of responding to lead.

 Extensive studies of cytoplasmic lead-binding proteins in nonlead-treated rats, human, and monkeys have been reported. Whether these proteins have formed as a result of environmental exposure to lead or are preformed is at present unclear. The lead-binding protein in rat kidney has been identified as a cleavage product of *α*2- microglobulin. The low molecular weight lead-binding proteins in human kidney have been identified as thymosin *β*4 (molecular weight 5 kDa) and acyl-CoA-binding protein (molecular weight 9 kDa). In human brain, the lead-binding proteins were thymosin *β*4 and an unidentified protein of 23 kDa. The principal remaining question is whether the p32/6.3 intranuclear lead-binding protein initially described by Shelton and Egle [2] represents a condensation product from these low molecular weight proteins.

## 12. Conclusions

Lead-binding proteins occur in many organs in the body, including kidney, brain, erythrocyte, liver, and lung. In most instances, they appear to be of low molecular weight, inducible by lead exposure, and to afford some protection against lead toxicity. The exception is the enzyme delta-aminolevulinic acid dehydratase (ALAD), which is present in high concentration in the erythrocyte, and although further inducible by lead exposure, is exquisitely sensitive to inhibition by lead. 

 The relationships between the intranuclear lead-binding protein of the kidney, the cytoplasmic lead-binding proteins, and the lead-binding proteins in erythrocytes remain to be determined. What is clear is that the inducible lead-binding proteins afford protection against lead toxicity.

## Abbreviations

ALAD: Delta-aminolevulinic acid dehydratase
ATPase: Adenosine triphosphatase
Ca^++^: Calcium ion
CaNa_2_EDTA: Calcium disodium ethylenediaminetetraacetic acid
Cd^++^: Cadmium ion
DMSA: Dimercaptosuccinic acid
Fe^++^: Iron ion
FPLC: Fast protein liquid chromatography
GFAP: Glial fibrillary acidic protein
GRP78: Glucose-regulated protein
ICP-MS: Inductively coupled plasma mass spectroscopy
i.p.: Intraperitoneal
*Kd*: Dissociation constant
kDa: Kilodalton
kg: Kilogram
M: Molar
mRNA: Messenger ribonucleic acid
Na-K-ATPase: Sodium-potassium-activated adenosine triphosphatase
Pb: Lead
SDS-PAGE: Sodium dodecyl sulfate polyacrylamide gel electrophoresis
UV: Ultraviolet
Zn^++^: Zinc ion.
